# Graphical Abstract: ChemistryOpen 2/2015

**DOI:** 10.1002/open.201580211

**Published:** 2015-04-15

*Chemistry Open* is a multidisciplinary, gold-road, open-access, international forum for the publication of Full Papers and Communications from all areas of chemistry and related fields. *ChemistryOpen* also publishes the Thesis Treasury containing summaries of Ph.D. theses and links to the full version via our homepage. Based in Europe, *ChemistryOpen* attracts authors and readers from around the world, as open-access publishing becomes more important in all areas of chemistry. *ChemistryOpen* is co-owned by ChemPubSoc Europe and published by Wiley-VCH. Authors can submit their primary research articles and thesis summaries via our homepage by clicking “submit an article”. All contributions considered suitable for publication are subject to peer review, and if accepted, electronically processed and published online ensuring high quality and short publication times.

## COVER PICTURE

**The cover picture shows** a *GreenCaps* microcapsule breaking and releasing encapsulated glycerol after the organosilica microspheres are sprayed from a pressurized polyurethane foam can. This shows how glycerol acts as a solid curing agent, promoting crosslinking of partially polymerized diphenylmethane diisocyanate. For more details, see the Full Paper on p. 120 ff.

**Figure:**
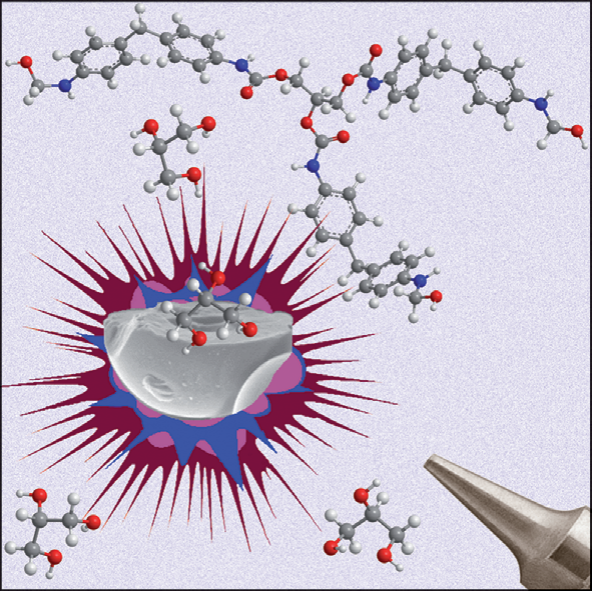


## COVER PROFILE

R. Ciriminna, A. Fidalgo, L. M. Ilharco,* M. Pagliaro*

Sol-Gel Microspheres Doped with Glycerol: A Structural Insight in Light of Forthcoming Applications in the Polyurethane Foam Industry

“*If you want to insulate or seal with polyurethane foam, instead of waiting two days for it to cure, you will be able to do it in a few hours using a foam can with our microcapsules.*” Learn more about the story behind the research featured on the front cover in this issue's Cover Profile. Read the corresponding article on p. 120 ff.

**Figure:**
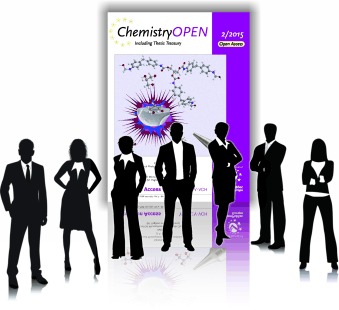


## NEWS

Spotlights on our sister journals 88–91

## COMMUNICATIONS

H.-K. Walter, P. R. Bohl.nder, H.-A. Wagenknecht*

92–96

Development of a Wavelength- Shifting Fluorescent Module for the Adenosine Aptamer Using Photostable Cyanine Dyes

**Split and recombine:** A DNA-based aptasensor for adenosine is described. The optical module is attached to the left side of the recognition module and combines a green emitting dye as an energy donor and a red emitting dye as an energy acceptor to allow fluorescent color readout of adenosine binding.

**Figure:**
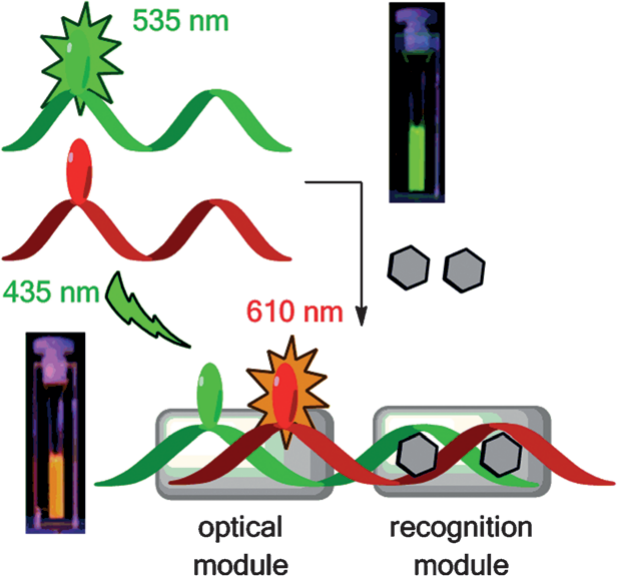


J.-F. Du, W. Li, L. Li, G.-B. Wen, Y.-W. Lin,* X. Tan*

97–101

Regulating the Coordination State of a Heme Protein by a Designed Distal Hydrogen-Bonding Network

**Fine-tuning heme proteins:** Heme coordination state determines the functional diversity of heme proteins. We introduced distal glutamic acid (Glu29) and histidine (His43) residues in myoglobin and regulated the heme into a non-native bis-His coordination state with native ligands His64 and His93, resembling natural globins such as cytoglobin and neuroglobin. This new approach can be generally applied for fine-tuning the structure and function of heme proteins.

**Figure:**
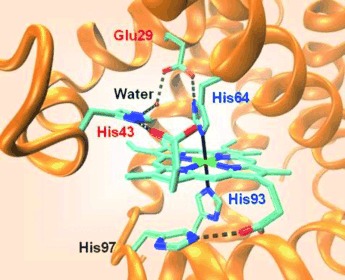


N. Iida, K. Tanaka, E. Tokunaga, H. Takahashi, N. Shibata*

102 –106

Regioisomer-Free C4h b-Tetrakis(tertbutyl) metallo-phthalocyanines: Regioselective Synthesis and Spectral Investigations

**A solid case for regioselectivity!** The C4h-selective synthesis of b-(tert-butyl)- metallophthalocyanines by tetramerization of a-trialkylsilyl phthalonitriles with metal salts following acid-mediated desilylation is disclosed for the first time. Investigation of regioisomer-free zinc btetrakis( tert-butyl)phthalocyanine using spectroscopy showed that the C4h single isomer is distinct in the solid state to zinc b-tetrakis(tert-butyl)phthalocyanine obtained by a conventional method.

**Figure:**
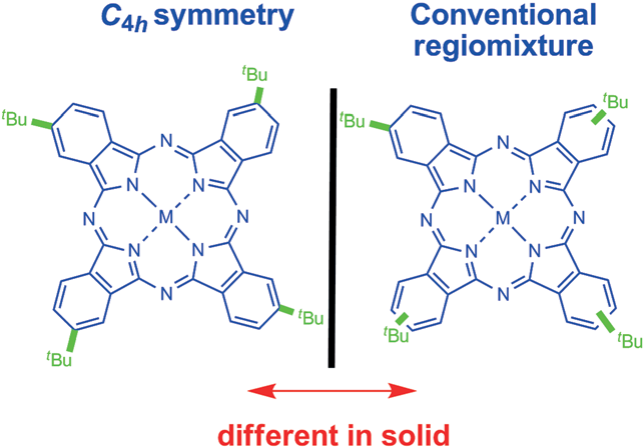


C. B. R. Reddy, S. R. Reddy,* S. Naidu

107 –110

Chemoselective Oxidation of Benzyl, Amino, and Propargyl Alcohols to Aldehydes and Ketones under Mild Reaction Conditions

**Chemoselective alcohol oxidation!** Catalytic oxidation reactions often suffer from low yields and selectivity. Here we develop a method using copper(I) catalysts to selectively oxidize a wide range of benzyl and propargyl alcohols to their corresponding aldehydes and ketones under mild reaction conditions and with excellent yields. This chemoselective catalytic oxidation scheme can tolerate even sensitive and oxidizable groups such as alkynes, amines, and phenols.

**Figure:**
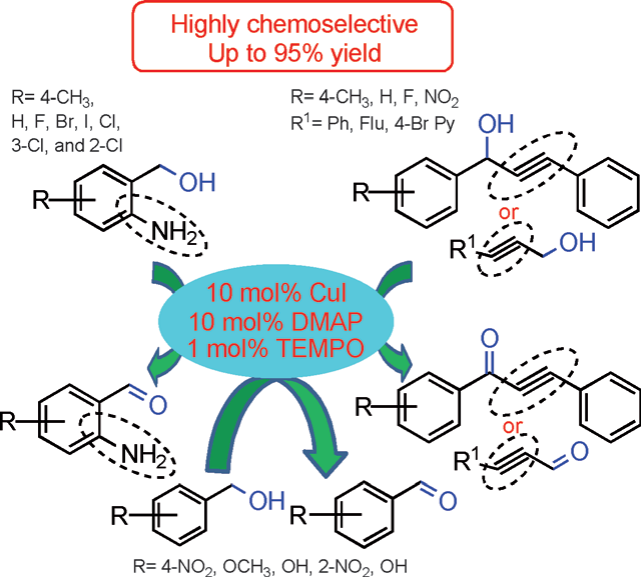


Y. Zhang, N. M. Magdaong, M. Shen, H. A. Frank, J. F. Rusling*

111–114

Efficient Photoelectrochemical Energy Conversion using Spinach Photosystem II (PSII) in Lipid Multilayer Films

**Powered like Popeye!** Spinach photosystem II (PSII) protein was used for the first time in lipid films to make a photoelectrochemical device. Photocurrents from PSII in a ∼2 mm biomimetic dimyristoyl- phosphatidylcholine (DMPC) film on a pyrolytic graphite (PG) anode were ∼20 mAcm¢2 in 40 mWcm¢2 light. When used in a photobiofuel cell with a platinum black mesh cathode, the PSII– DMPC photoanode gave an output voltage of 0.6 V and a maximum output power 14 mWcm¢2.

**Figure:**
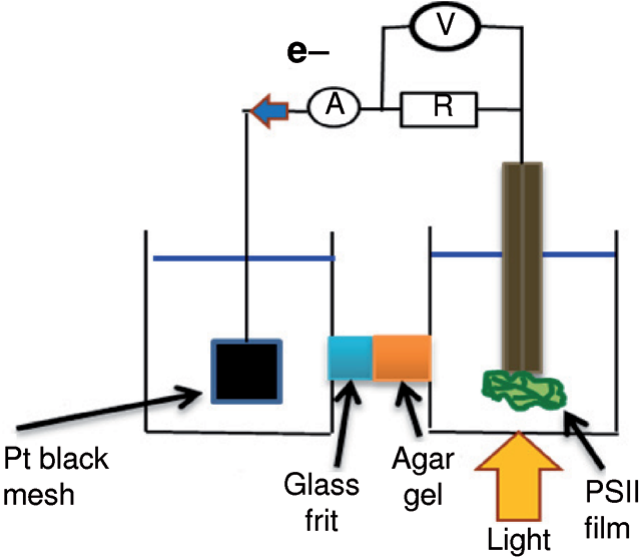


E. Cunha, M. F. ProenÅa, F. Costa, A. J. Fernandes, M. A. C. Ferro, P. E. Lopes, M. Gonz.lez-Debs, M. Melle-Franco, F. L. Deepak, M. C. Paiva*

115– 119

Self-Assembled Functionalized Graphene Nanoribbons from Carbon Nanotubes

**Ribbons from tubes:** Pyrrolidine-functionalized graphene nanoribbons were observed to assemble into few-layer stacks. The interlayer distance was measured by transmission electron microscopy and X-ray diffraction, and calculated by computer modelling, to be approximately 0.5 nm. The nanoribbons were obtained by unzipping of functionalized carbon nanotubes.

**Figure:**
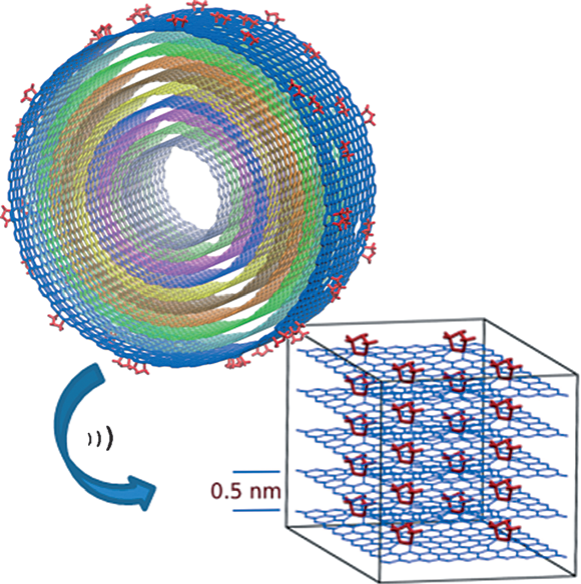


## FULL PAPERS

R. Ciriminna, A. Fidalgo, L. M. Ilharco,* M. Pagliaro*

120 –126

Sol-Gel Microspheres Doped with Glycerol: A Structural Insight in Light of Forthcoming Applications in the Polyurethane Foam Industry

**Blasting organosilica microspheres:** Porous silica-based microspheres containing glycerol can be curing agents for foams. An easy and scalable sol-gel process was used to make organosilica microspheres doped with glycerol. The structure reveals glycerol is efficiently encapsulated, acts as a template, barely leaches, but is released by depressurization. These microspheres can later be used as high-quality environmentfriendly solid curing agents for spray polyurethane foams.

**Figure:**
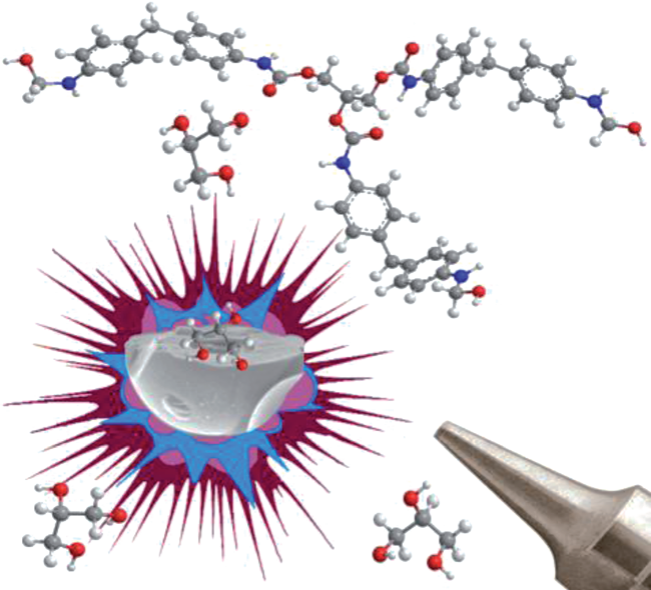


O. Penon, T. PatiÇo, L. Barrios, C. Nogu¦s, D. B. Amabilino, K. Wurst, L. P¦rez-Garc.a*

127 –136

A New Porphyrin for the Preparation of Functionalized Water-Soluble Gold Nanoparticles with Low Intrinsic Toxicity

**The power of gold!** A new thiolated dissymmetrical porphyrin was synthesized and consequently immobilized onto gold nanoparticles using the Brust–Schiffrin method. Thiolated polyethylene glycol was added to obtain water-soluble nanoparticles. The nanoparticles could be internalized by cells and were nontoxic. Tests on the ability of the functionalized gold nanoparticles to induce singlet oxygen production point to a promising nanosystem for photodynamic therapy.

**Figure:**
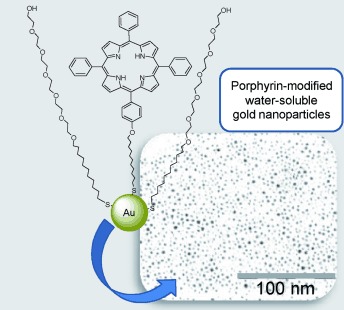


B. Balcomb, M. Singh, S. Singh*

137 –145

Synthesis and Characterization of Layered Double Hydroxides and Their Potential as Nonviral Gene Delivery Vehicles

**Nonviral gene delivery:** Layered double hydroxides (LDHs) exhibit anionexchange chemistry making them suitable carriers of DNA. Mg¢Al, Mg¢Fe, Zn¢ Al, and Zn¢Fe compounds known to be LDHs were synthesized and found to bind DNA in varying degrees. Nuclease digestion studies revealed the LDHs afford partial protection to bound DNA. HEK293 cells were successfully transfected using the LDHs, and minimal toxicity was observed, showing their potential as viable alternatives to other nonviral gene delivery systems.

**Figure:**
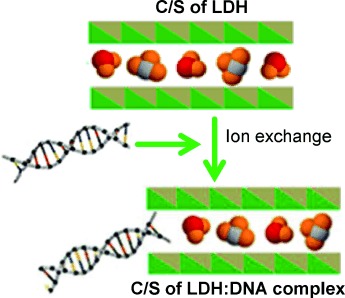


T. N. Ravishankar, T. Ramakrishnappa,* G. Nagaraju,* H. Rajanaika

146 –154

Synthesis and Characterization of CeO2 Nanoparticles via Solution Combustion Method for Photocatalytic and Antibacterial Activity Studies

**Nanoparticles for the environment:** CeO2 nanoparticles are competent photocatalysts for environmental applications because of their strong redox ability, nontoxicity, stability, and low cost. We synthesized CeO2 nanoparticles using a solution combustion method. These show photocatalytic activity in the degradation of trypan blue (a typical pollutant dye), antibacterial activity against Pseudomonas aeruginosa, and detoxifying activity as shown in the reduction of Cr^VI^ to Cr^III^.

**Figure:**
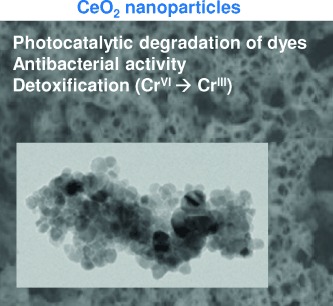


R. Losantos, M. S. Churio, D. Sampedro*

155 –160

Computational Exploration of the Photoprotective Potential of Gadusol

**Sunscreen computed:** Gadusol is one of the simplest natural UV-absorbing compounds in aquatic organisms. A detailed CASPT2//CASSCF theoretical study describes the underlying features responsible for the photoprotective capacity of gadusol, which very efficiently dissipates light energy as heat. This study provides insight for the design of new synthetic sunscreens.

**Figure:**
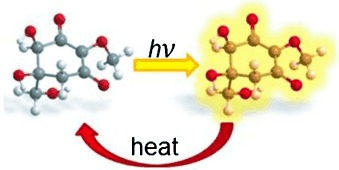


E. Cadoni,* G. Ferino, P. Pitzanti, F. Secci, C. Fattuoni, F. Nicolý, G. Bruno*

161 –168

Halogen and Hydrogen Bonding Benzothiophene Diol Derivatives: A Study Using ab initio Calculations and X-Ray Crystal Structure Measurements

**Halogen be thy bond:** X-ray crystal structures and ab initio calculations reveal that bromo- (top) and iodobenzothiophene diols (bottom) can generate intermolecular interactions with p electrons and/or with oxygen atoms through halogen bonding.

**Figure:**
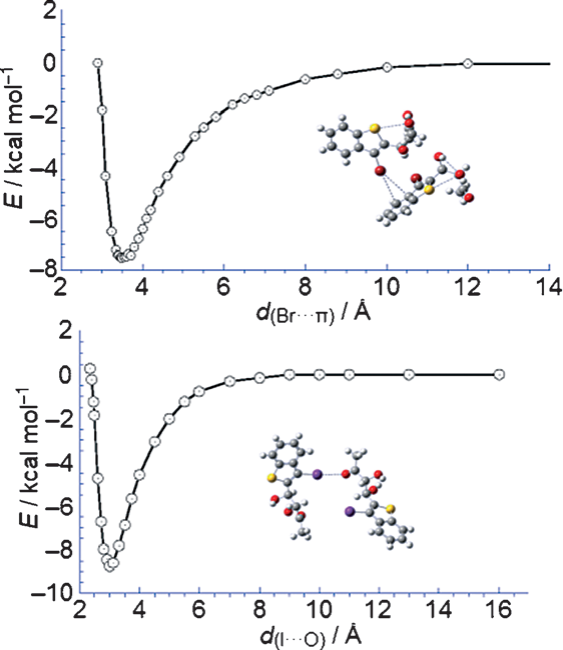


S. Estalayo-Adri.n, R. Lartia, A. Meyer, J.-J. Vasseur, F. Morvan, E. Defrancq*

169 –173

Assessment of the Full Compatibility of Copper(I)-Catalyzed Alkyne-Azide Cycloaddition and Oxime Click Reactions for bis-Labelling of Oligonucleotides

**All roads lead to Rome!** A new procedure for the efficient bis-conjugation of oligonucleotides through successive oxime ligation (Click-O) and copper(I)- catalyzed alkyne–azide cycloaddition (Click-H) or vice-versa is reported starting from 5’-amino, 3’-diol-functionalized oligonucleotide as an easily accessible precursor. The Click-O followed by Click- H route was found to be more efficient for accessing the bis-labelled oligonucleotide than the reverse.

**Figure:**
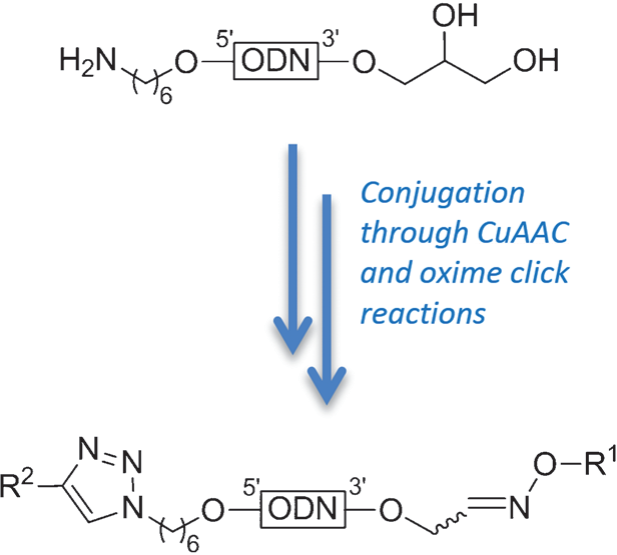


J. Strand, P. Nordeman, H. Honarvar, M. Altai, A. Orlova, M. Larhed, V. Tolmachev*

174 –182

Site-Specific Radioiodination of HER2- Targeting Affibody Molecules using 4-Iodophenethylmaleimide Decreases Renal Uptake of Radioactivity

**Reducing renal radioactivity!** Affibody molecules are small scaffold-based affinity proteins with promising properties as probes for radionuclide-based molecular imaging. The use of the more lipophilic 125I-4-iodophenethylmaleimide (IPEM) instead of 125I-3-iodo-((4-hydroxyphenyl) ethyl)maleimide (IHPEM) for sitespecific radioiodination of Affibody molecules leads to a twofold decrease in renal retention of radioactivity.

**Figure:**
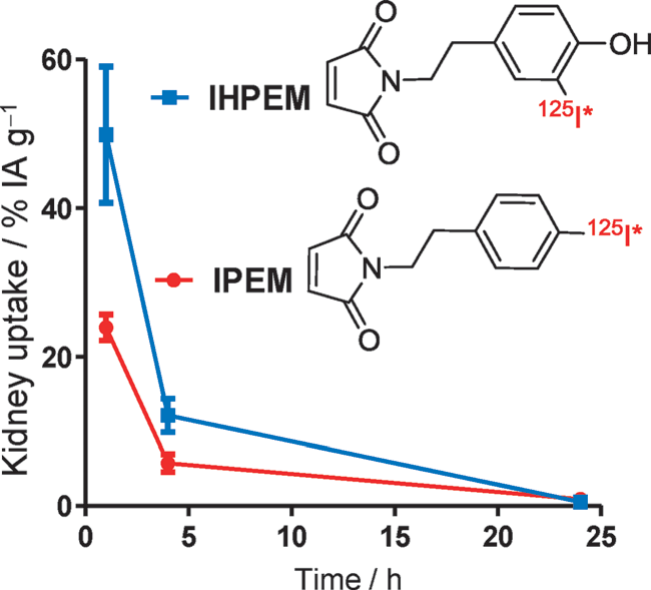


C. Nardon, F. Chiara, L. Brustolin, A. Gambalunga, F. Ciscato, A. Rasola, A. Trevisan, D. Fregona*

183 –191

Gold(III)–pyrrolidinedithiocarbamato Derivatives as Antineoplastic Agents

**Gold versus cancer!** We report here on the synthesis, physico-chemical characterization, and solution behavior of two gold(III) pyrrolidinedithiocarbamates (PDT): [AuIIIBr2(PDT)] and [AuIIICl2(PDT)], with the bromide compound being more cytotoxic towards different cancer cell lines. A chemical and biological evaluation of the bromide compound shows it is able to trigger a ROS cascade, which possibly lead to apoptosis from the opening of the permeability transition pore.

**Figure:**
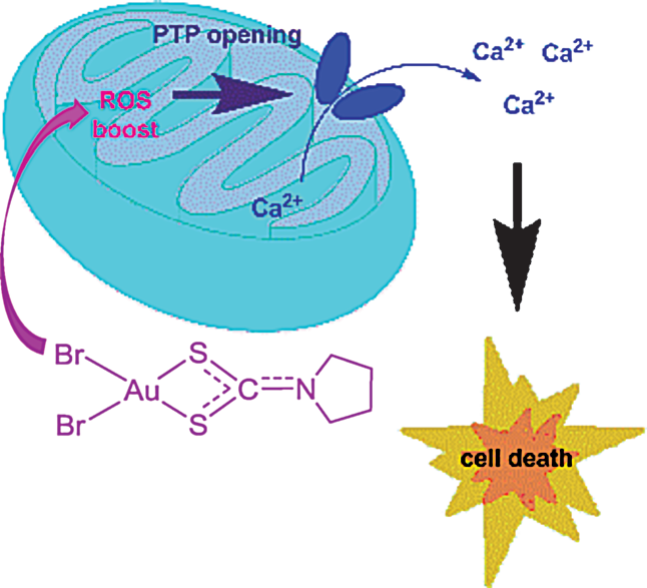


## THESIS TREASURY

C. Bhat*

192 –196

Synthetic Studies of Alkaloids Containing Pyrrolidine and Piperidine Structural Motifs

**Avenues to asymmetric alkaloids!** Various 2-substituted pyrrolidine and piperidine chiral bioactive natural products were synthesized using a ‘chiral pool’ method. l-proline and l-pipecolinic acids with one chiral center served as the best precursors for the synthesis of these alkaloids. Overall, 14 total synthetic and 11 formal synthetic approaches were developed.

**Figure:**
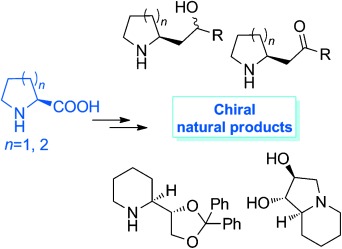


## SERVICE

* Author to whom correspondence should be addressed.

Supporting information is available on the WWW (see article for access details).

A video clip is available as Supporting Information on the WWW (see article for access details).

This is an open-access article, published under the terms and conditions of a Creative Commons License, as stated in the final article.

Contributions labeled with this symbol have been judged as “Very Important Papers” by the referees.

